# Comparison of Microstructure and Mechanical Properties of Ultra-Narrow Gap Metal Active Gas Arc Welded and Narrow Gap Submerged Arc Welded Q235A Low Carbon Steel

**DOI:** 10.3390/ma16196601

**Published:** 2023-10-09

**Authors:** Shang Wu, Wenkai Xiao, Lingfei Gong, Fuju Zhang

**Affiliations:** 1 School of Power and Mechanical Engineering, Wuhan University, Wuhan 430072, China; ws13526073335@163.com (S.W.); glf17396155202@163.com (L.G.); 2 Wuhan Narogap Intelligent Equipment Co., Ltd., Wuhan 430072, China; fjzhang@whu.edu.cn

**Keywords:** ultra-NGMAGW, NGSAW, AF, microstructure evolution

## Abstract

The 18 mm thick Q235A low carbon steel plates were welded via the ultra-narrow gap metal active gas arc welding (ultra-NGMAGW) and narrow gap submerged arc welding (NGSAW), and the microstructure and mechanical properties of the welded joints’ area were characterized. The results showed that there is acicular ferrite (AF) in the weld zone of the joint obtained via the ultra-NGMAGW. The AF grains are fine and have a great difference in growth direction, resulting in high local dislocation density. However, there is no AF in the welded joint obtained via the NGSAW. Using numerical simulation analysis of the temperature field distribution and the thermal cycle curve in the welding process of the ultra-NGMAGW, it was found that the mechanism of microstructure evolution is that during the welding process of the ultra-NGMAGW, the heat input is low, the cooling rate is quick, and the residence time in the high temperature region is short. Therefore, pearlite with coarse grains is basically not formed. AF nucleates in different directions with inclusions as the core. The tensile strength of the weld joint obtained via the ultra-NGMAGW is 643 MPa, which corresponds to 139% of that of the base metal, and 132% of that obtained via the NGSAW. The ultra-NGMAGW joints exhibited better tensile strength and higher microhardness than the NGSAW joints, which is mainly due to the existence of AF.

## 1. Introduction

Low carbon steel refers to steel with a carbon content between 0.05 and 0.25%, which has low strength and hardness, good plasticity, and toughness. Due to its good processability and cost effectiveness, low carbon steel is used in a wide variety of applications such as manufacturing, construction, automotive, and energy industries. In these industries, it is often necessary to weld low carbon steel thick plates. In traditional welding technology, such as submerged arc welding (SAW), it owns a high welding cladding rate and a convenient welding process, but the removal of welding slag is difficult, and for the large welding thermal deformation in SAW, the heat treatment process must be increased to eliminate it after welding. At the same time, the heat input in the welding process is large, which may promote grain coarsening, resulting in the decline of the welded joints. And, Li et al. [1] found that in SAW, the heat-affected zone (HAZ) is usually brittle and weak because the coarse austenite grains and poor microstructure are formed in the welding process.

Narrow gap welding (NGW) is a process to complete multi-layer and multi-pass welds in a much narrower gap than the conventional welding groove width. It has the characteristics of high efficiency and high quality for welding thick plates. Due to the increased production of thick plate welded structures, NGW technology was popularized in many fields in the early 1980s, such as petrochemical high-pressure machines, power plant boiler thick-walled cylinders, nuclear reactor vessels, turbine shafts, and large steel structures. The ultra-narrow gap welding (ultra-NGW) method further reduces the gap of the weld, which greatly reduces the cross-sectional area of the welding and simplifies the groove processing, and also reduces the cost of welding materials and power consumption. The arc current used in the welding process is less dense and generates less heat input, thus reducing the risk of sheet deformation and residual stress. Compared with traditional welding technology, NGW can reduce the plastic and toughness damage to the heat-affected zone and increase the service life. At the same time, the thick plate adopts a narrow and deep welding groove, which can reduce the stomata defects. The HAZ is narrow and the grains obtained are fine, which improves the welding quality. Considering the small groove angle and small gap, how to ensure side-wall fusion, improve fusion welding formation, and prevent weld cracks is a challenge faced by the ultra-NGW [2].

Zhang et al. [3] developed four technologies including the multi-functional integrated ultra-narrow gap mixed gas arc welding (ultra-NGMAGW) gun, adaptive intelligent tracking of the weld trajectory, efficient gas protection of the high-temperature welding zone, and high stability of the droplet transition, which put ultra-NGMAGW welding technology into production. At present, Zhang et al. have successfully welded some typical steel such as 35Cr2Ni2Mo [4] and 35CrMoV [5] using ultra-NGMAGW, and their research has shown that ultra-NGMAGW can significantly improve the strength of the weld. It is because the ultra-NGMAGW has a lower groove filling area and less heat input than the NGSAW, which further reduces the impact of high temperature on the HAZ during welding and ensures the performance of the welded joint.

A large number of research experiments have shown that the test steel containing AF has satisfactory mechanical properties [6,7,8,9]. At the same time, Li et al. [10] found that there were AF with fine grains in the welded joints of the NGW and characterized the mechanical properties, and then found that the strength of the welded joints was higher than that of the bulk material and it had good plasticity. In this paper, low carbon steel Q235A was selected as the welding object, and the ultra-NGMAGW and the NGSAW welding experiments were carried out. We characterized the microstructure and mechanical properties of the welded joints under the two welding methods and obtained the temperature field distribution and thermal cycle curve in the ultra-NGMAGW via numerical simulation, accordingly, and analyzed the mechanism of tissue evolution and strengthening.

## 2. Materials and Methods

**Figure 1:**
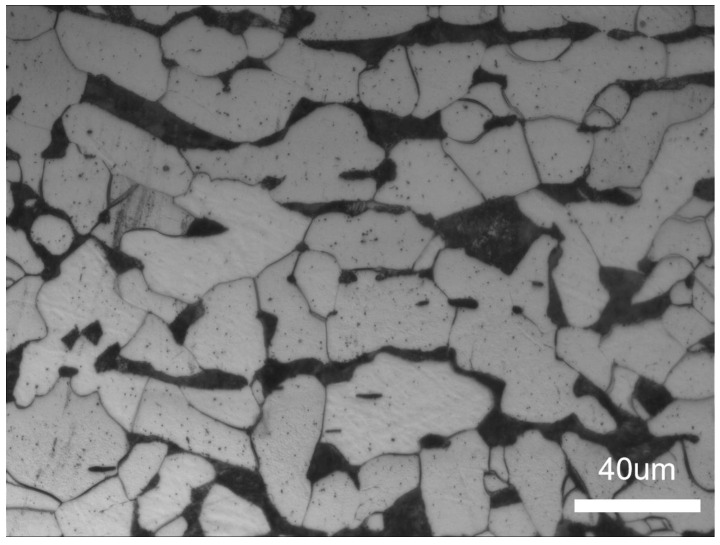
Microstructure of Q235A base metal.

The base material (BM) used in this study is a Q235A low carbon steel with a dimension (length × width × thickness) of 216 × 150 × 18 mm, respectively. The chemical composition of the BM is shown in Table 1, and all concentrations are defined in weight percentage. The microstructure of the BM after corrosion with the 3% nitrate alcohol solution is shown in Figure 1, which is a mixture of pearlite and ferrite. The filler wire used in this study is ER90S-G welding wire with a diameter of 1.2 mm, and the chemical composition of the welding wire is shown in Table 2.

**Figure 2:**
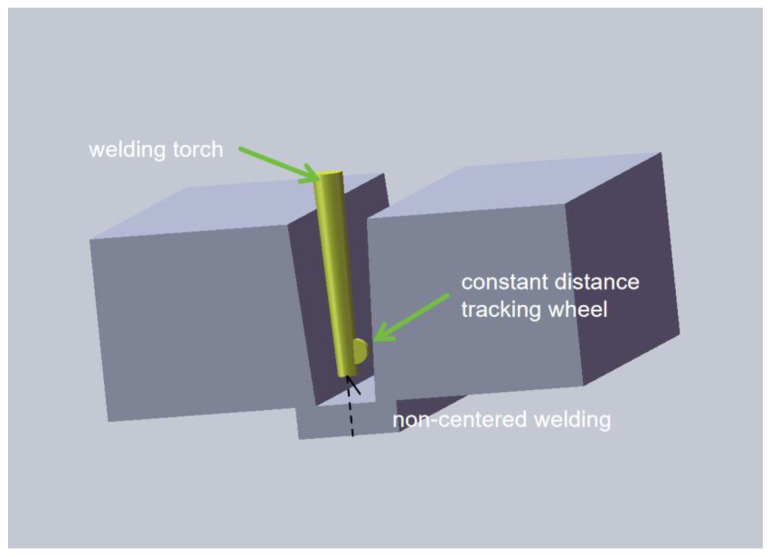
Schematic diagram of the ultra-NGMAGW device.

**Figure 3:**
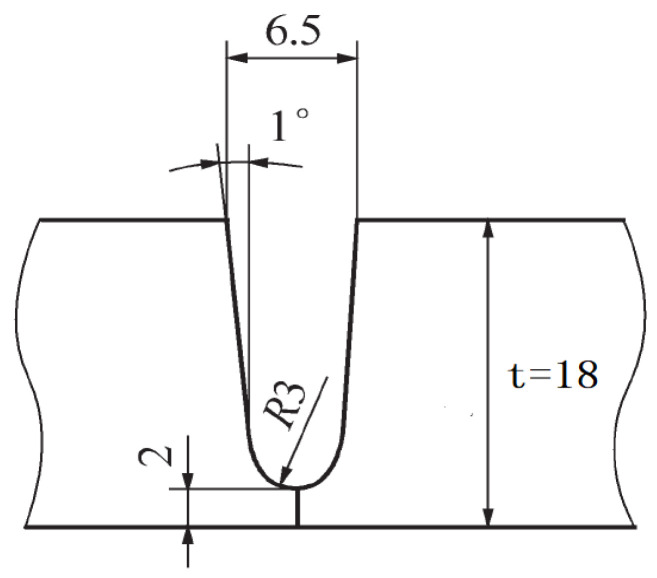
Schematic diagram of the ultra-NGMAGW welding groove.

The equipment used in this ultra-NGMAGW experiment is a six-axis cross-section of the ultra-narrow gap automatic welding equipment. The schematic diagram of the experimental device is shown in Figure 2. Special ceramic insulation components are installed at the end of the welding gun near the high-temperature area. The welding gun body is made of metal materials with good conductivity and thermal conductivity, and technical measures such as self cooling water circulation and efficient heat dissipation are adopted inside the body to ensure the reliability and stability of the welding process under NG-GMAW conditions.Reduce spatter and improve the droplet transition stability by precisely adjusting the ratio of mixed gases (Ar, CO_2_, O_2_, and He) using a gas-shielded welding power supply with a high quality cooperative control function. Based on contact sensing, the welding gun is designed to float between states to carry out adaptive weld tracking based on the elastic pressure balance principle. The schematic diagram of the groove after processing is shown in Figure 3. This groove is designed to make the blunt edge of the root of the weld more transparent and not to produce non-fusion of the side wall during welding.

Two 216 × 75 × 18 mm Q235A thick plates were joined with the above groove, and the area to be welded was polished to remove the oxide and impurities to improve the welding quality. The clamp was used to hold the base material for the ultra-NGMAGW. The detailed parameters of the ultra-NGMAGW are shown in Table 3.

After welding, the welded specimens were sectioned transverse to the welding direction and then mounted, ground, polished, and etched with a 3% Nital solution. In this study, the CarlZeissAxioLab.A1 optical microscope (OM) (Carl Zeiss, Oberkochen, Germany) and the TESCANMIRA3LMH mode field-emission scanning electron microscope (SEM) (TESCAN, Brno, Czech Republic) were used to characterize the microstructure of the specimens, and the grain size was measured from the OM image via an area equivalence. Electron back scattering diffraction (EBSD) was also used to characterize the grain size, orientation, and dislocation density in the weld zone. EBSD was performed using a detector field emission SEM with an acceleration voltage of 20 KV and a minimum scanning step size of 20 nm. The specimens were analyzed using HKL channel5 software 5.12.74.0. For measuring the grain size via EBSD, the grain was defined using a misorientation larger than 2° and the average grain size was calculated via an area equivalence.

Microhardness was measured from the weld zone to the base material zone of each sample with a load of 100 g and duration of 10 s using an HXV-1000A microhardness tester (Laizhou Lyric Testing Equipment Co., Ltd., Laizhou, China), and the indentation interval was set to 0.24 mm. All specimens were prepared using standard polishing procedures. The microhardness tester was calibrated with the standard specimens.

**Figure 4:**
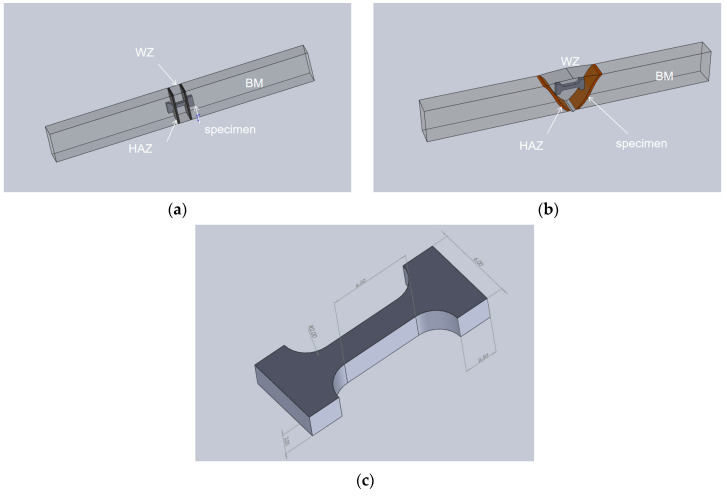
Cut position and size of tensile specimen: (**a**) sampling position of the ultra-NGMAGW; (**b**) sampling position of the NGSAW; and (**c**) sample size (mm).

For the uniaxial tensile test, 2 mm thick specimens with geometry and dimensions as shown in Figure 4 were cut from the selected area of the weld via electron-discharging machining. All specimens were polished with 1000 mesh sandpaper and then cleaned and dried with anhydrous ethanol to minimize the surface roughness. Tensile tests were conducted at room temperature on a universal testing machine (MTS E45) at a loading speed of 0.5 mm/min. Both specimens were tested at least three times.

**Figure 5:**
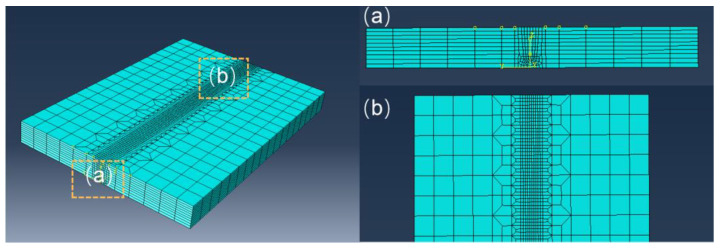
Q235A thick plate joint finite element model. (**a**) weld cross-section; (**b**) weld surface.

In order to study the formation of AF in the ultra-NGMAGW, we use the finite element analysis software Abaqus to simulate the temperature field distribution of Q235A low carbon steel during the ultra-NGMAGW. First, construct the Q235A thick plate model, which is sized to 216 × 150 × 18 mm, and then divide the model into hexahedral elements. The welding model is shown in Figure 5. And, the number of nodes and the number of grids is 21,759 and 19,656, respectively.

According to the heat input characteristics of the ultra-NGMAGW, the double ellipsoid heat source model is selected for simulation. The heat flux distribution equation of the double ellipsoidal heat source model is divided into the front hemisphere and the back hemisphere, as shown in Equation (1).

(1) $$\begin{array}{cc} & q_{1}\left(x,y,z,t\right)=\frac{6\sqrt[{}]{3}f_{1}Q}{a_{1}bc\pi \sqrt[{}]{\pi }}e^{-3(x+vt{)}^{2}/a_{1}{\;}^{2}}e^{-3y^{2}/b^{2}}e^{-3z^{2}/c^{2}}\\ & q_{2}\left(x,y,z,t\right)=\frac{6\sqrt[{}]{3}f_{2}Q}{a_{2}bc\pi \sqrt[{}]{\pi }}e^{-3(x+vt{)}^{2}/a_{2}{\;}^{2}}e^{-3y^{2}/b^{2}}e^{-3z^{2}/c^{2}} \end{array},$$

where *Q* = ƞP, ƞ is the thermal efficiency, *P* is the laser power, *v* is the welding speed, *t* is the welding time, *a*_1_, *a*_2_, *b*, *c* are the geometric parameters of the double ellipsoid heat source model, and *f*_1_ and *f*_2_ are the power distribution coefficients of the front and back semi-ellipsoid, respectively, generally *f*_1_ + *f*_2_ = 2. The welding heating process is a nonlinear transient heat conduction process which satisfies Fourier’s law, and the temperature field calculation formula is shown in Equation (2).

(2) $$\rho \left(T\right)\frac{\partial T}{\partial t}=Q\left(x,y,z,t\right)+\frac{\partial \left(\lambda \left(T\right)\frac{\partial T}{\partial x}\right)}{\partial x}+\frac{\partial \left(\lambda \left(T\right)\frac{\partial T}{\partial y}\right)}{\partial y}+\frac{\partial \left(\lambda \left(T\right)\frac{\partial T}{\partial z}\right)}{\partial z}\;\;\;\;$$

where $Q\left(x,y,z,t\right)$ is the internal heat source intensity, T is the temperature, t is the time, ρ is the material density, $\lambda \left(T\right)$ is the thermal conductivity of the material, and ∂T is the specific heat capacity of the material. In addition, it is necessary to determine the initial conditions and boundary conditions in the welding process. When t = 0, the initial temperature of the weldment is 20 °C. The boundary condition is the heat exchange between the surface of the test plate and the surrounding environment. For the analysis of the welding thermal process, the third type of boundary condition is considered, as shown in Equation (3):

(3) $$\;\lambda \frac{\partial T}{\partial n}=h\left(T_{w}-T_{f}\right)\;\;\;$$

where λ is the thermal conductivity of the object, $T_{w}$ is the surface temperature of the object, $T_{f}$ is the temperature of the medium around the object, and h is the heat transfer coefficient between the surface of the object and the surrounding medium. According to the actual welding conditions, the temperature of the medium around the weldment is set at 20 °C. In this paper, the convection heat transfer coefficient is defined as 18 × 10^−6^ W/(mm^2^·°C), and the solid line temperature is 1450 °C. Due consideration is given to the role of thermal radiation, and the radiative rate is defined as 0.8.

The ultra-NGMAGW involves multiple-pass filled bevelings. The upper pass does not have more additional heat input than the lower pass, while the lower pass is subject to additional heat cycling due to the filling of the upper pass. This simulation adopts the death and death unit technology. After the beginning, the elements of the first weld will be awakened and generate heat, and the subsequent elements will be awakened one after another as the heat source moves. After the simulation of the first weld is completed, the calculation of the next weld will be awakened, and the above procedure will be repeated until the simulation of the three welds is completed.

## 3. Results

### 3.1. Structure and Morphology of Welded Joints

**Figure 6:**
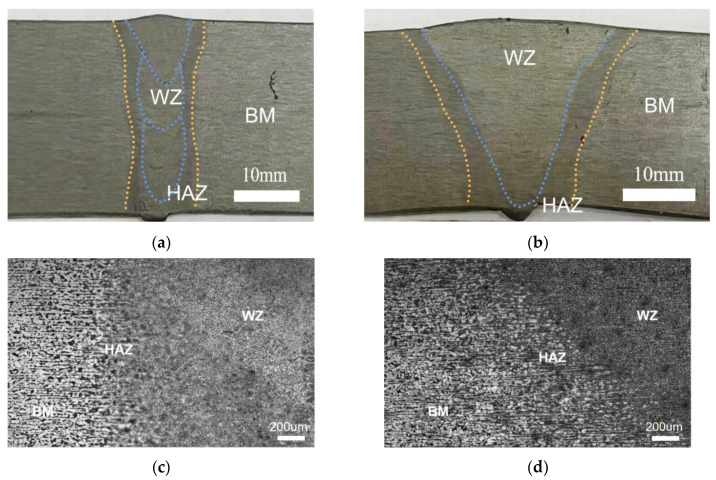
Cross-section profiles and its 50× enlarged view of Q235A welded joints: (**a**) cross-section profiles of Ultra-NGMAGW; (**b**) cross-section profiles of NGSAW; (**c**) enlarged view of Ultra-NGMAGW; and (**d**) enlarged view of NGSAW.

The cross-section profiles of the welded joints of 18 mm thick Q235A thick plates after the ultra-NGMAGW and the NGSAW are respectively shown in Figure 6. The ultra-NGMAGW specimens showed a “U” shape fusion zone, while the NGSAW specimens showed a “V” shape fusion zone. The Ultra-NGMAGW joint can be clearly distinguished via three weld paths.

**Figure 7:**
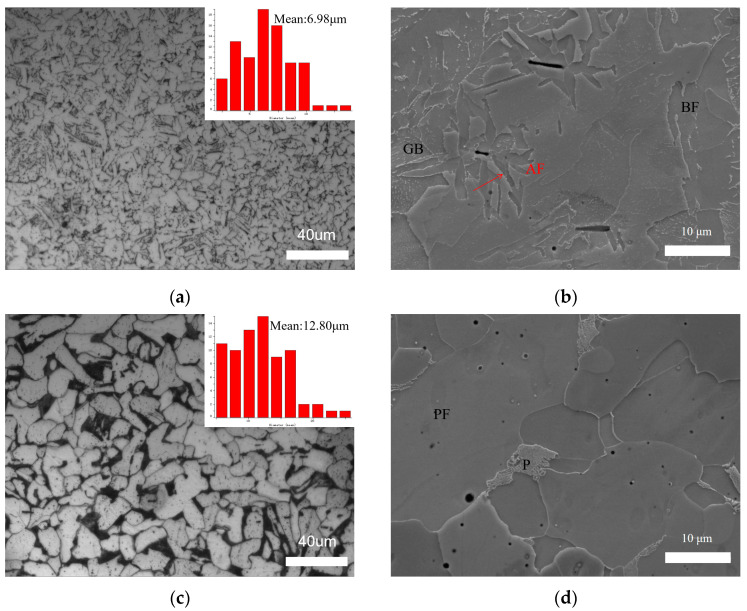
Microstructure of Q235A weld zone: (**a**) OM of ultra-NGMAGW; (**b**) SEM of ultra-NGMAGW; (**c**) OM of NGSAW; and (**d**) SEM of NGSAW.

The microstructure of the weld zone (WZ) obtained via the ultra-NGMAGW and NGSAW are presented in Figure 7. Figure 7a,b shows the ultra-NGMAGW WZ microstructure, mainly composed of acicular ferrite (AF), bulk ferrite (BF), and a small fraction of granular bainite (GB). Figure 7c,d shows the microstructure of the NGSAW WZ joint, mainly composed of proeutectoid ferrite (PF) and pearlite (P). However, as shown in Figure 7a,c, it can be found that the weld grain obtained via the ultra-NGMAGW is finer than that obtained via the NGSAW. The mean grain size of the ultra-NGMAGW welded joints is about 6.98 μm, while it is 12.80 μm in the NGSAW welded joints. In addition, Figure 7b shows that the AF grows in all directions around with inclusions or defects as the core [11,12], and in the growth process, due to the different nucleation directions or large growth angles of AF in the adjacent grains, cross-collision occurs to form a locking mechanism, which hinders the continued growth of the grains. In Figure 7d, only P and PF with a coarse grain structure were observed, while AF does not exist. In addition, we can find more dispersed carbides existing in the ultra-NGMAGW joints compared to the NGSAW joints.

**Figure 8:**
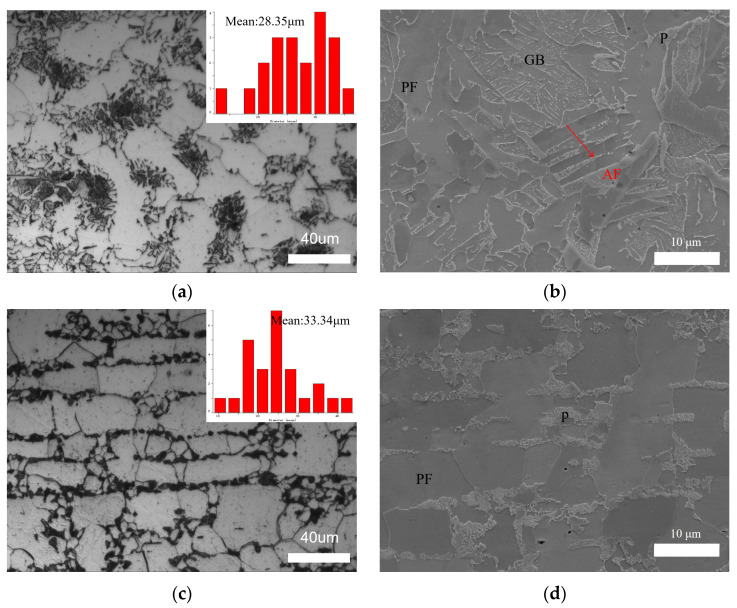
Microstructure of Q235A HAZ: (**a**) OM of ultra-NGMAGW; (**b**) SEM of ultra-NGMAGW; (**c**) OM of NGSAW; and (**d**) SEM of NGSAW.

The microstructure of the heat-affected zone (HAZ) obtained via the ultra-NGMAGW and the NGSAW is shown in Figure 8. Figure 8a,b shows the microstructure of the HAZ of the ultra-NGMAGW sample, mainly composed of GB, BF, and a small amount of AF and P. Figure 8c,d are the microstructures of the HAZ of the NGSAW sample, mainly composed of PF and P. Compared with Figure 8a,c, it can be found that the HAZ grains obtained via the ultra-NGMAGW are finer than those obtained via the NGSAW. The mean grain size of the ultra-NGMAGW HAZ is about 28.35 μm, while it is 33.34 μm in the NGSAW HAZ. This is mainly due to the fact that, as shown in Figure 8b, a small amount of AF and GB are interwoven into the tissues obtained from the ultra-NGMAGW, while coarse grain PF in the tissues are obtained from the NGSAW as shown in Figure 8d, and a large amount of pearlite exists between the ferrites.

In summary, it can be found that the microstructure of both the WZ and HAZ obtained via the ultra-NGMAGW is more uniform and finer than that obtained via the NGSAW, which is mainly due to the presence of AF in the joint obtained via the ultra-NGMAGW.

### 3.2. Mechanical Properties 

#### 3.2.1. Microhardness 

**Figure 9:**
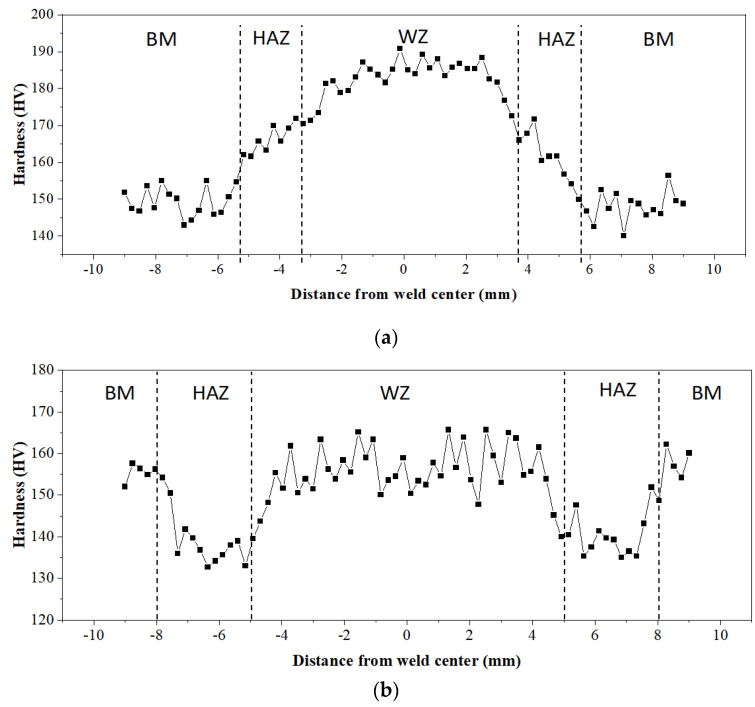
Microhardness measurements on the welded joint (WZ: weld center; HAZ: heat-affected zone; BM: base material): (**a**) ultra-NGMAGW, and (**b**) NGSAW.

The Vickers hardness values of the Q235A welded joints were determined after the ultra-NGMAGW and the NGSAW were respectively measured via the HVS-1000A digital microhardness tester (Laizhou Lyric Testing Equipment Co., Ltd., Laizhou, China). The hardness maps of the entire weld for both the ultra-NGMAGW and NGSAW joints are shown in Figure 9a,b, respectively. Due to the hardness variation in different zones, the WZ, HAZ, and BM can be clearly distinguished from the hardness contour map.

The results show that the average hardness of the BM region of both the two specimens is about 155HV0.1, while the average hardness in the WZ and HAZ regions of the ultra-NGMAGW specimen is higher than that of the NGSAW specimen. The average hardness in the WZ for the ultra-NGMAGW sample is about 185HV0.1, and in the HAZ, it is about 165HV0.1. Obviously, the peak hardness is located in the WZ. The NGSAW sample has a hardness of 155HV0.1 in the WZ and a hardness of 140HV0.1 in the HAZ.

By comparing Figure 9a,b, it can be found that there is a certain degree of softening in the joint tissue of the NGSAW; this may be due to the precipitation ferrite, as shown in Figure 8d, induced by the thermal welding cycle in the area of the HAZ and the fusion line as well as the presence of soft ferrite, which leads to the microhardness drop in these regions. There is no such phenomenon in the ultra-NGMAGW. According to Figure 7 and Figure 8, the increase in hardness in the WZ is related to the generation of AF, while the increase in hardness in the HAZ is the result of a large amount of GB structure transformation and a small amount of AF.

#### 3.2.2. Tensile Property

**Figure 10:**
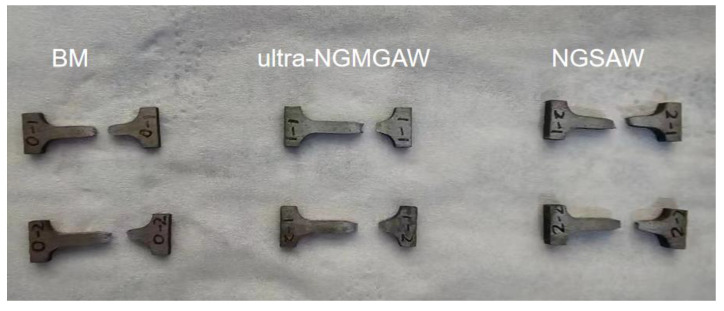
Tensile specimen after fracture.

Tensile tests were conducted on the Q235A welded joints of the ultra-NGMAGW and the NGSAW, respectively, and the fractured samples are shown in Figure 10. It can be seen that the ultra-NGMAGW joint failed in the BM well away from the weld, which indicates that the Q235A did not weaken after the ultra-NGMAGW, while the NGSAW joint failed at the weld, which indicates that the NGSAW joint was not as strong as the BM.

**Figure 11:**
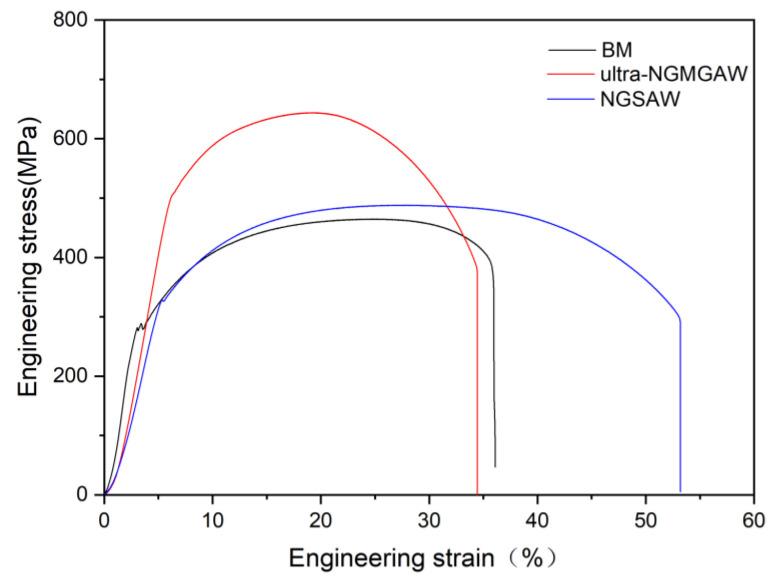
Representative engineering stress versus engineering strain curves for BM, and Ultra-NGMAGW and NGSAW Q235A joints.

Figure 11 shows the representative engineering stress versus the engineering strain curves for the BM, and the ultra-NGMAGW and NGSAW Q235 steel welded joints. The details of the transverse tensile test results are given in Table 4. The yield strength (YS), ultimate tensile strength (UTS), and apparent elongation for the ultra-NGMAGW joints were obtained as 504 MPa, 643 MPa, and 34.5%, respectively, and the NGSAW specimens demonstrated a YS of 327 MPa, a UTS of 487 MPa, and an apparent elongation of 53.2%, respectively. While the YS, UTS, and apparent elongation of the BM were 281 MPa, 464 MPa, and 36.1%, respectively. The Ys of both the two kinds of welded joints are apparently higher than the BM. However, under the premise that the elongation is close to the BM, the Ultra-NGMAGW joint performs more excellent tensile strength, which corresponds to 139% of that of the base metal, and 132% of that obtained via the NGSAW. And, the NGSAW joints performs very high elongation.

**Figure 12:**
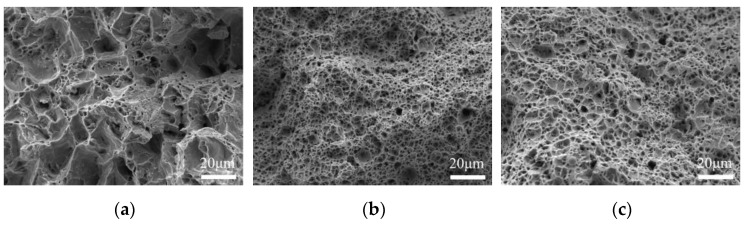
SEM micrographs of the tensile fracture surfaces of (**a**) BM; (**b**) ultra-NGMAGW; and (**c**) NGSAW.

The fracture surface morphology of the welded joints obtained via SEM is depicted in Figure 12. High magnification observations reveal that all the ductile fracture regions present dimples and microvoids, with some of the dimples containing spherical inclusions, so they all show a ductile fracture mode. While the dimples in the BM are obviously bigger and deeper from Figure 12a, the dimples in the ultra-NGMAGW are finer and more uniform. This also explains that the welded joints obtained via the ultra-NGMAGW own the highest tensile strength.

Using the above experimental results, it can be found that the WZ and the HAZ of the welded joint obtained via the ultra-NGMAGW has higher strength and hardness. According to Xiao et al. [13], the microstructure characteristics of AF can improve the strength and toughness of the steel to a certain extent, which is consistent with the observed microstructure in Figure 7 and Figure 8. It can be considered that the strengthening in the ultra-NGMAGW joint comes from AF.

## 4. Discussion

### 4.1. Reinforcement Mechanism

In previous analyses, we have known that the excellent mechanical properties of the welded joints obtained via ultra-NGMAGW are mainly due to the formation of AF, which in turn produces grain refinement in the welded joint structure. In order to further analyze the effect of the ultra-NGMAGW welding method on the crystal’s characteristics and reveal the relationship between the welding method and the mechanical properties, the specimens after the ultra-NGMAGW and NGSAW were analyzed via EBSD.

**Figure 13:**
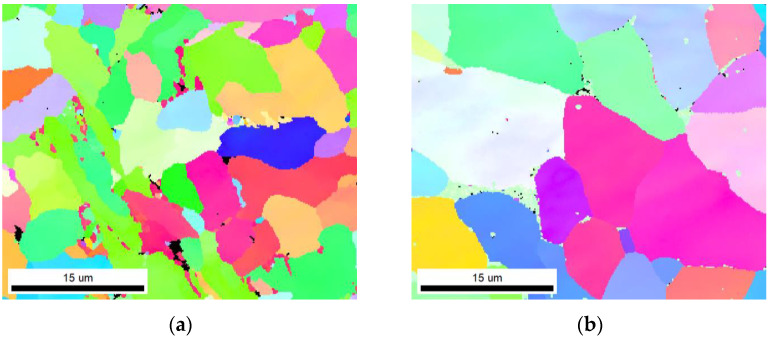

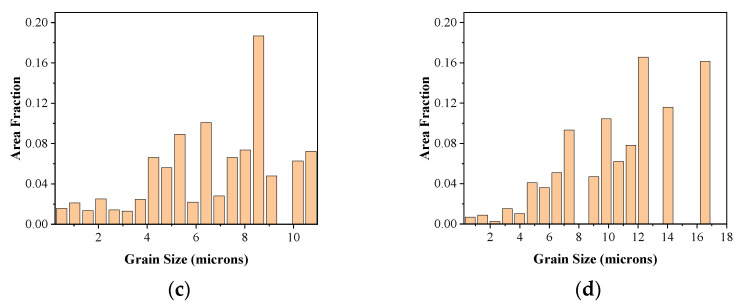
Inverse pole figure and grain size distribution diagram at the WZ: (**a**) inverse pole figure of the ultra-NGMAGW joint; (**b**) inverse pole figure of the NGSAW joint; (**c**) grain size distribution diagram of the ultra-NGMAGW joint; and (**d**) grain size distribution diagram of the NGSAW joint.

Figure 13 shows the WZ microstructure inverse pole figure and corresponding grain size distribution diagram obtained via ultra-NGMAGW and NGSAW, respectively. Figure 13a,b shows the inverse pole of two types of welding microstructures. According to the statistical results of the grain size in Figure 13c,d, it can be found that the average grain size of the WZ structure obtained via the ultra-NGMAGW is about 7 μm, which is basically consistent with the aciculate ferrite size found in the study [14], and the average grain size of the WZ structure obtained via NGSAW is 11 μm. Obviously, the grains obtained via the ultra-NGMAGW is finer.

**Figure 14:**
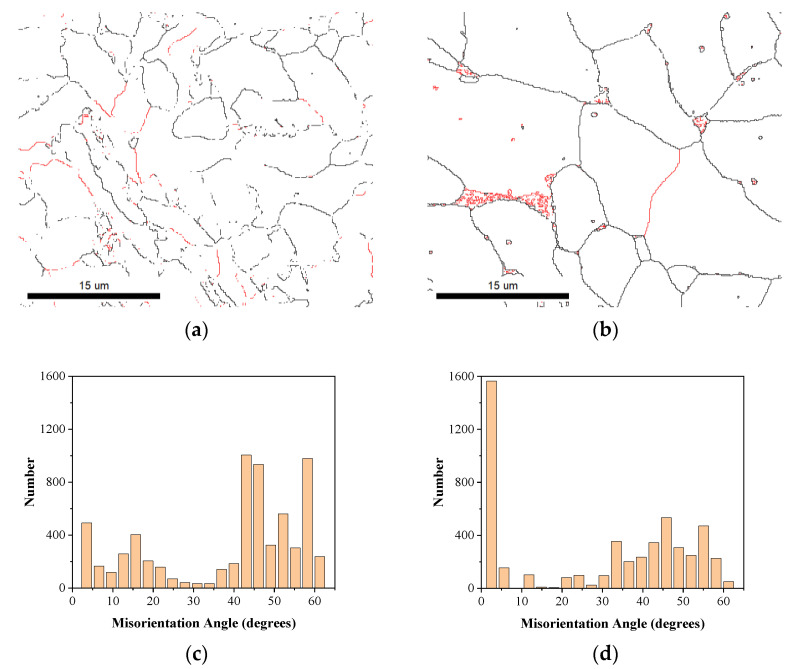
Adjacent grain boundary misorientation distribution map and angle statistical map at the WZ: (**a**) adjacent grain boundary misorientation distribution map of ultra-NGMAGW joint; (**b**) adjacent grain boundary misorientation distribution map of the NGSAW; (**c**) angle statistical map of the ultra-NGMAGW; and (**d**) angle statistical map of the NGSAW.

Figure 14 shows the grain boundary characteristic distribution diagram at the WZ of the two welding methods and the statistical diagram of the misorientation angle of adjacent grain boundaries. The low-angle boundary with adjacent grain misorientation between 2–15° are marked with red lines, and the high-angle boundary with adjacent grain misorientation greater than 15° are marked with black lines. According to the grain boundary characteristic distribution and the grain boundary orientation distribution results, the number of high-angle boundaries in the weld of the specimens after ultra-NGMAGW is significantly higher than that after NGSAW. In previous work [15], high-angle boundaries can inhabit crack propagation and change its direction, so the strength of the ultra-NGMAGW welded joints are obviously higher than the NGSAW.

**Figure 15:**
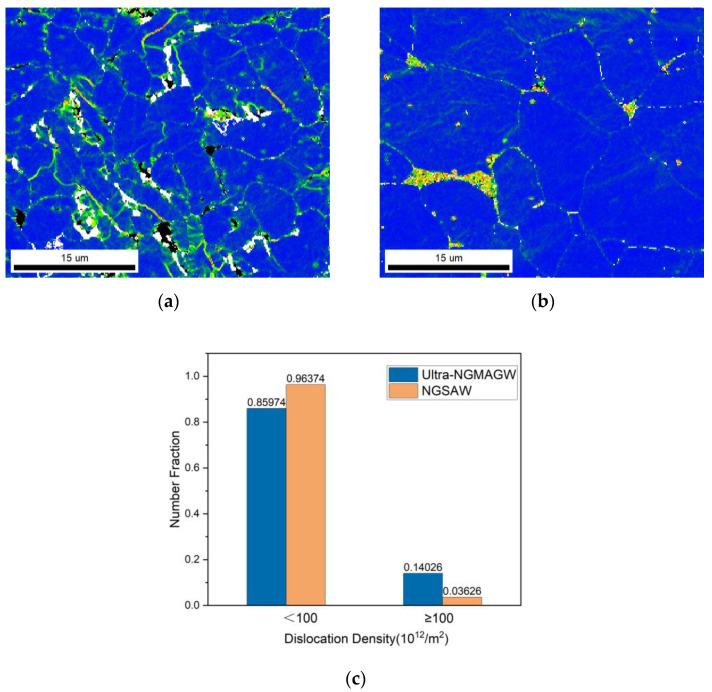
The distribution diagram of the KAM and statistical diagram of dislocation density in the WZ: (**a**) the KAM of the ultra-NGMAGW; (**b**) the KAM of the NGSAW; and (**c**) the statistical diagram of dislocation density in the ultra-NGMAGW and NGSAW.

The distribution of grain boundary misorientation in the weld microstructure can reflect the magnitude of dislocation density inside the weld microstructure. Figure 15 shows the kernel average misorientation diagram of two types of welding microstructures, and the kernel average misorientation (KAM) is defined as the misorientation between each data point and its neighboring points.

As we know from Figure 7, the ultra-NGMGAW WZ joint is mainly composed of AF and BF, while the NGSAW WZ joint is mainly composed of BF and P. There are only a few high-density dislocation regions at the junction of BF. As can be seen from the local misorientation results in Figure 15c, the proportion of weld dislocation density above 100 obtained via the ultra-NGMGAW can reach nearly 15%, while the proportion of weld dislocation density above 100 obtained via the NGSAW is only close to 5%. The dislocation density in the region of the AF distribution is significantly higher than that of the BF distribution. AF has a high density of dislocation inside, so when the welded joint is deformed via external force, it is easy to emit a dislocation at the tip of the crack, forming a packet structure of the dislocation and the timely passivation of the crack so that the crack growth is blocked.

In summary, the welded joints obtained via the ultra-NGMAGW own higher strength than that of the NGSAW, which is mainly due to the AF obtained during the ultra-NGMAGW. As we know, there are a large number of dispersed carbide phases in the ultra-NGMAGW playing a dispersion strengthening role, which has also been studied in previous work [16]. The grain size obtained of the ultra-NGMAGW weld joints is smaller than that of the NGSAW. According to the Hall–Petch relationship [17], the welded joints obtained via the ultra-NGMAGW own higher strength, which reflects the effect of the fine grain strengthening of AF corresponding to Figure 10 and Figure 14. In addition, the smaller the grain size, the greater the number of high-angle boundaries. At the same time, due to the smaller grain size in the Ultra-NGMAGW, it increases the mean volume fraction of grain boundaries, which may act as barriers and provide obstacles to the propagation of brittle cracks [15,18], so grain boundary strengthening is also reflected in the ultra-NGMAGW. And, in general, the high angle grain boundaries effectively resist the crack propagation and change its direction [19]. The number of high-angle boundaries in the weld of the specimens after the ultra-NGMAGW is significantly higher than that after the NGSAW, so the effect of grain boundary strengthening is more obvious in the ultra-NGMAGW joints. Additionally, as we know, when the dislocation density in steel is relatively large, it will hinder the slip of the dislocation and improve the strength of the steel. It is precisely because of the presence of AF that the weld obtained via the ultra-NGMAGW can improve the mechanical properties of the weld under the co-action of fine grain strengthening, grain boundary strengthening, and dislocation strengthening.

### 4.2. Numerical Simulation and Microstructure Evolution

The mechanical properties of the welded metal are closely related to their microstructures, which are dependent on the chemical composition of the material and the thermal history (cycles) due to the welding processes [20,21].

According to Figure 7 and Figure 8, we found that in the ultra-NGMAGW, the pearlite and ferrite in the BM undergo structural transformation, where GB and a small amount of AF appear in the HAZ, while the pearlite changes to a large amount of AF and a few GB in the WZ. AF is the main factor affecting the mechanical properties of the welded joints.

**Figure 16:**
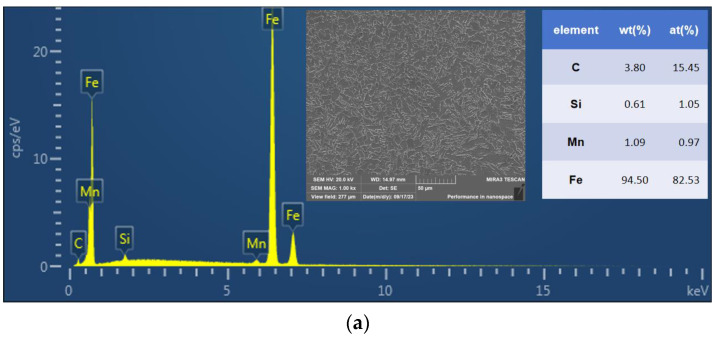

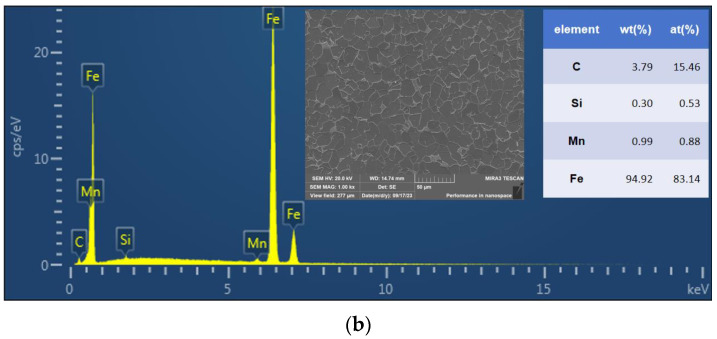
(**a**) EDS spectrum for the ultra-NGMAGW welded joints, and (**b**) EDS spectrum for the NGSAW welded joints.

As seen in Figure 16a,b, the EDS results of the welded joints obtained via the ultra-NGMAGW and the NGSAW are shown. The content of Si and Mn in the specimens of the ultra-NGMAGW is significantly higher than that of the NGSAW. The alloying elements in steel promote the nucleation and refinement of AF [22]. That is to say, the higher composition of Si and Mn in the welded joints of the ultra-NGMAGW promotes the formation of AF, which in turn optimizes the mechanical properties of the ultra-NGMAGW joints.

**Figure 17:**
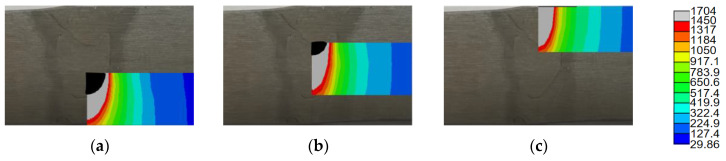
Temperature distribution of welded joint: (**a**) first weld; (**b**) second weld; and (**c**) third weld.

The weld temperature distribution results of the Q235A ultra-NGMAGW joints obtained via finite element simulation are shown in Figure 17. The simulated temperature field distribution shape diagram of the weld is in good agreement with the positions of the weld zone and the heat-affected zone in the actual welding process, indicating that the simulation is effective.

**Figure 18:**
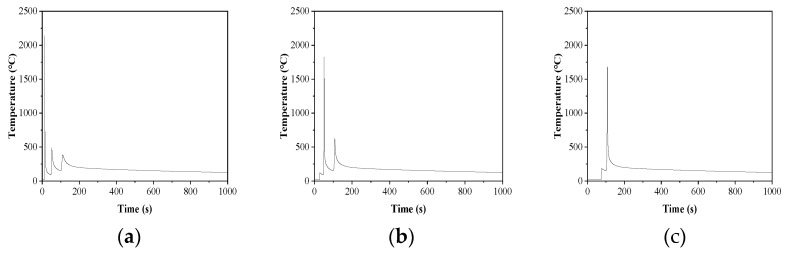
Welding thermal simulation of thermal cycle curve in the weld zone: (**a**) the first weld; (**b**) second weld; and (**c**) third weld.

The temperature at the center of the three passes is selected as the temperature of the WZ and the thermal cycle curve is obtained, which is shown in Figure 18.

Figure 18a shows that during the first welding, the temperature of the weld zone of the weld bead rapidly rose to 2140.3 °C, and then rapidly decreased to 152.5 °C. After that, the second welding was performed in Figure 18b. This time, the temperature of the weld zone of the weld bead rose to 1827.6 °C, and the first weld was heated to 488 °C, and then quickly decreased to 152.2 °C. For the third welding in Figure 13c, the temperature of the weld in this pass was increased to 1684.4 °C, and the first two welds were heated to a maximum of 387.0 °C and 639.2 °C, respectively, and then rapidly cooled to 115.2 °C after 1000 s.

It can be seen from the thermal cycle curve of the weld zone that due to the small heat input during the welding process, the temperature of the weld zone will drop rapidly after rising to the maximum height and will not stay on the Ac3 line for too long. In addition, the subsequent pass welding will not heat the previous pass temperature above the Ac1 line, so the influence of the subsequent pass on the microstructure evolution of the previous pass can be ignored.

According to studies [23,24], various types of medium temperature transition structures will be generated during the continuous cooling of low-carbon steel. Granular bainite structures are mainly obtained at a low cooling rate, and lath bainite structures are mainly obtained at a high cooling rate. During continuous cooling, AF can be formed in the higher transition temperature range.

Combined with the weld thermal cycle curve in Figure 18, and the SEM micrographs in Figure 7b, it can be concluded that austenite grains will transform into different ferrite structures during the process of the temperature drop after the ultra-NGMAGW. First of all, a very small part of austenite is transformed into pearlite during the rapid cooling process, and then transforms to bulk ferrite as the temperature drops. As the temperature drops to about 600 °C, more AF nucleates at the inclusion [25], and granular bainite appears after continuous cooling.

While in the NGSAW, combined with the SEM micrographs in Figure 7d and due to the high heat input and low cooling rate of the NGSAW, pearlite and bulk ferrite with coarse grains will be formed, and these coarse grains will reduce the strength and toughness of the weld.

## 5. Conclusions

(1) AF exists in the microstructure of the ultra-NGMAGW welded Q235A joints, and the grain size of AF is smaller than that of pearlite and bulk ferrite in the NGSAW welded joints.

(2) The welded joints obtained via the ultra-NGMAGW have better mechanical properties, and the hardness and strength are higher than those obtained via the NGSAW.

(3) The main reason for the formation of AF is that the heat input in the welding process is small, the cooling rate is fast, and the high temperature residence time is short.

(4) AF with a small grain size, high-angel boundary, and high dislocation density exists in the joints obtained via the ultra-NGMAGW, which results in excellent deformation resistance and mechanical properties under the co-action of fine grain strengthening, grain boundary strengthening, and dislocation strengthening mechanism.

## Figures and Tables

**Table 1:** Chemical composition of Q235A steel (wt%).

C	Si	Mn	S	P	Nb	Fe
0.14–0.22	0.30	0.3–0.65	0.05	0.04	0.15–0.16	Bal.

**Table 2:** Chemical composition of ER90S-G (wt%).

C	Si	Mn	S	Cr	Ni	Mo	V	W	Cu	Fe
0.06	0.40	0.78	0.001	2.68	0.06	0.01	0.21	1.69	0.04	Bal.

**Table 3:** Welding process parameters of the ultra-NGMAGW.

Arc Voltage	Welding Current	Welding Speed	Protective Gas	Gas Flow
26.4 V	195 A	4.64 mm/s	80% (Ar) + 20% (CO_2_)	20 L/min

**Table 4:** Tensile properties for the ultra-NGMAGW, NGSAW, and BM.

	Yield Strength (MPa)	Tensile Strength (MPa)	Elongation (%)
BM	281	464	36.1
ultra-NGMAGW	504	643	34.5
NGSAW	327	487	53.2

## Data Availability

Data available on request due to restrictions eg privacy or ethical. The data presented in this study are available on request from the corresponding author.
